# Modulation of Mesangial Cells by Tamsulosin and Pioglitazone Under Hyperglycemic Conditions: An In Vitro and In Vivo Study

**DOI:** 10.3390/ijms26199277

**Published:** 2025-09-23

**Authors:** Sandra Lizbeth Aguilera-Martínez, Martín Humberto Muñoz-Ortega, Sandra Luz Martínez-Hernández, Jorge Christopher Morones-Gamboa, Javier Ventura-Juárez

**Affiliations:** 1 Department of Morphology, Basic Sciences Center, Universidad Autónoma de Aguascalientes, Aguascalientes 20100, Mexico; al140436@edu.uaa.mx (S.L.A.-M.); al239857@edu.uaa.mx (J.C.M.-G.); 2 Department of Chemistry, Basic Sciences Center, Universidad Autónoma de Aguascalientes, Aguascalientes 20100, Mexico; humberto.munozo@edu.uaa.mx; 3 Department of Microbiology, Basic Sciences Center, Universidad Autónoma de Aguascalientes, Aguascalientes 20100, Mexico; luz.martinez@edu.uaa.mx

**Keywords:** fibrosis, high glucose, inflammation, mesangial cell, tamsulosin, pioglitazone, mesangial expansion

## Abstract

Diabetic nephropathy (DN) is a consequence of diabetes mellitus (DM), in which hyperglycemia triggers osmotic and oxidative stress and activates inflammatory pathways. These processes damage kidney cells, with mesangial cells (MCs) undergoing mesangial expansion. Antihyperglycemic drugs prevent the progression of renal disease. Although tamsulosin is not conventionally used for the treatment of DN, its previously reported anti-fibrotic and anti-inflammatory effects in liver and lung injury models suggest that it may exert renoprotective actions like those of pioglitazone, which has also been shown to improve cellular carbohydrate and lipid metabolism. MCs were exposed to 20 mM glucose medium and treated with either 50 nM tamsulosin or 100 nM pioglitazone. Subsequently, cell proliferation, inflammatory markers (NF-κB, IL-1β, IL-17), fibrogenic markers (TGF-β, collagen I), oxidative stress parameters (NRF2, superoxide), and indicators of mesangial activation (α-SMA, rhodamine–phalloidin) were assessed in vitro. Both treatments reduced cellular proliferation and hypertrophy, attenuated the release of reactive oxygen species (ROS), decreased IL-17 and α-SMA expression, and reduced mesangial activation and hypertrophy. In an in vivo model of DN in Wistar rats, both treatments decreased mesangial cell activation and expansion. In conclusion, tamsulosin and pioglitazone exert anti-fibrogenic and anti-inflammatory effects in MCs exposed to HG, thereby limiting mesangial activation and expansion.

## 1. Introduction

DN is a complication of DM which affects approximately 40% of patients with type 2 DM (T2DM) and 30% of those with type 1 DM (T1DM). DN is associated with increased morbidity and mortality, primarily due to microvascular events [1,2,3]. It is one of the leading causes of chronic kidney disease (CKD) and, in advanced stages, may progress to end-stage renal disease (ESRD) [4,5].

Hyperglycemia is the primary sign presented in DM, which causes complications by disrupting metabolic and hemodynamic homeostasis. It has been estimated that between 25% and 40% of patients with DM develop DN after 10 to 35 years of disease progression [6].

DN is characterized by glomerular basement membrane (GBM) thickening, mesangial expansion with or without nodular sclerosis, podocyte death, and endothelial disruption, followed by a decrease in the number of nephrons [7]. DN progresses through three stages: first, glomerular hypertrophy and glomerulus hyperfiltration; then, tubulointerstitial inflammation and hypertrophy or glomerular expansion; finally, a reduction in cell number due to apoptosis and mesangial matrix accumulation in advanced stages [8].

On the other hand, metabolic alterations and bioproducts generated during glucose metabolism activate various signaling pathways, leading to increased ROS production [9]. The major activated pathways include those related to the formation of advanced glycation end-products (AGEs), which produce cell damage [10]; the polyol pathway, in which glucose is converted to sorbitol, resulting in both osmotic and oxidative stress [11,12]; and the hexosamine pathway, through which glucose generates O-GlcNAcylated products that modify transcription factors such as specificity protein (Sp1), thereby enhancing Sp1-dependent gene expression and promoting the synthesis of both TGF-β and plasminogen activator inhibitor 1 (PAI-1) [13,14].

MCs, which comprise approximately 30–40% of the glomerular cell population [15], have been shown to express three types of glucose transporters: SGLT2 and GLUT-4 (insulin-sensitive facilitative transporters), as well as GLUT-1, the most abundant and functionally significant transporter [12,16,17]. Under hyperglycemic conditions, GLUT-1 overexpression has been demonstrated to induce TGF-β1 expression, which promotes mesangial matrix production and, in a chronic state, inhibits its proliferation [12,18].

Mesangial expansion is the consequence of three important events: first, the proliferation of MCs, followed by excessive production of mesangial matrix and, finally, MC hypertrophy [19]. Initially, MCs undergo proliferation as part of the response to kidney damage, contributing to the initial state of mesangial expansion [19,20]. Afterwards, the effect of TGF-β is linked to phenotypic MC trans-differentiation to the myofibroblast phenotype, resulting in increased protein synthesis [20,21].

The normal mesangial matrix is composed of collagens IV and VI, laminin, fibronectin, thrombospondin, and chondroitin sulfate proteoglycans [22]. Activation of MCs leads to the expression of proteins that are normally absent in mesangial matrix and are difficult to eliminate, including collagens I and III (interstitial/fibrillar); notably, collagen I [23,24] and fibronectin are major components of fibrotic tissue. Additional factors that induce mesangial expansion include TGF-β, platelet-derived growth factor (PDGF), IL-1β, tumor necrosis factor α (TNF-α), basic fibroblast growth factor (bFGF), angiotensin II (ANG II), connective tissue growth factor (CTGF), insulin-like growth factor 1 (IGF-1), eicosanoids, and leptin [25].

Finally, the hypertrophy of MCs is characterized by decreased proliferation, α-SMA overexpression, and an augmented polygonal-shaped morphology [19].

It is challenging to achieve safe and effective responses to pharmacological treatments for CKD at present, as inflammation is not a secondary phenomenon but a central mechanism that perpetuates the progression of renal damage even when drugs are administered. The sustained activation of cytokines and profibrotic factors maintains signaling pathways that continue to drive disease progression independently of the therapeutic effect [26,27]. Therefore, the first-line treatment consists of hyperglycemia control drugs, among which SGLT2 inhibitors (iSGLT2) and glucagon-like peptide-1 (GLP-1) agonists stand out [28]. Both types of drugs are used to decrease proteinuria and hypertensive nephropathy; however, these treatments only manage to delay disease progression and often require high doses to achieve therapeutic effects, which may increase the risk of adverse events [26,27].

Furthermore, it is important to highlight reports emphasizing the significance of β-AR signaling in mitochondrial biogenesis and its roles in the regulation of glucose metabolism, inflammation, and apoptosis in the kidney. In various pathological contexts such as DN, consideration of β-AR signaling suggests promising directions for the pharmacological and molecular modulation of β-adrenergic pathways as a therapeutic strategy against renal diseases [29].

Tamsulosin, a sulfonamide and a selective antagonist of α1A- and α1D-AR, is mainly used to relieve symptoms associated with benign prostatic hyperplasia (BPH). Furthermore, it is employed for the treatment of nephrolithiasis, ureteral calculi, prostatitis, and various urinary dysfunctions [30]. In an in vitro model of DN with glomerular endothelial cells, tamsulosin has been shown to reduce pro-inflammatory cytokines such as IL-1β, IL-6, TNF-α, and IL-8, as well as TGF-β1 and collagen I expression [31].

On the other hand, pioglitazone acts as an insulin sensitizer and helps to control hyperinsulinemia. It is administered to patients diagnosed with T2DM as a second or third-line drug, either as monotherapy or in combination with metformin or sulfonylureas [30,32].

Although few studies have focused on the use of pioglitazone as an anti-fibrotic, the activation of PPAR-γ receptors has been found to reduce proliferation and apoptosis in ANG II-activated MCs [33,34,35]. In an in vivo study conducted by Ko et al. in 2008 [36], it was found that administration of pioglitazone in T2DM rats had a renoprotective effect by inhibiting NF-κB activation and decreasing TGF-β expression and collagen synthesis, which ameliorated DN. These results are in agreement with those reported by Maeda et al. in 2005, who performed a similar in vitro study in TGF-β1-activated MCs [37].

Furthermore, in 2014 Wang et al. activated MCs in vitro in a medium with HG concentration (25 mM), observing increased ROS production levels through the expression of p22phox and p47phox. Upon treatment of these cells with pioglitazone, a decrease in the expression of these markers was observed in a dose-dependent manner, suggesting that it could act as a modulator of oxidative stress; however, its mechanism of action remains unknown [38].

In addition to its anti-inflammatory effects, researchers have begun to investigate its antihypertensive effects, which indirectly contribute to the reduction in renal damage due to the large number of PPAR-γ receptors expressed in the mesangium. In fact, it has been observed that the presence of PPAR-γ agonists yields better results than suppression of the activity of the renin–angiotensin system using angiotensin-converting enzyme inhibitors [34].

Finally, in an in vivo DM model used in our laboratory involving Wistar rats induced with streptozotocin, it was observed that tamsulosin and pioglitazone reduced the damage caused by elevated serum glucose levels, acting as renoprotective agents and restoring the morphological structure. Furthermore, our research group has reported improvements in markers of renal function, including serum creatinine, urea, BUN, and FBG levels; reduced expression of inflammatory markers such as NF-κB and IL-1β was reduced; increased IL-10 levels; and a decrease in fibrosis along with lower expression of TGF-β and collagen IV [39]. Therefore, the objective of this study was to evaluate the effects of tamsulosin and pioglitazone on MCs (SV40 MES 13) exposed to HG concentrations in vitro, in order to determine whether these drugs have significant effects on the oxidative, inflammatory, and fibrogenic responses of these cells that are key in the development of DN and, consequently, the mesangial expansion observed in the in vivo model.

## 2. Results

### 2.1. Impacts of HG, Tamsulosin, and Pioglitazone on Viability and Cellular Integrity of SV40 MES 13 Cells

**Figure 1:**
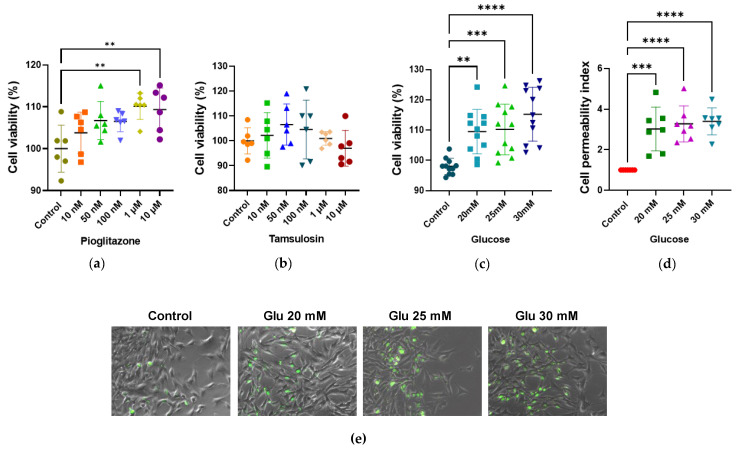
Viability of SV40 MES 13 cells at different concentrations of glucose, pioglitazone, and tamsulosin. (**a**) Cell viability in SV40 MES 13 cells treated with pioglitazone and (**b**) tamsulosin at concentrations of 10 nM, 50 nM, 100 nM, 1 μM, and 10 μM, compared to untreated cells. (**c**) SV40 MES 13 cells treated with HG at concentrations of 20 mM, 25 mM and 30 mM, compared to the control. Cell viability was detected using MTT assay. (**d**,**e**) Cell permeability was detected using Sytox Green assay. The image was taken with ×200. Statistical significance is indicated as follows: ** *p* < 0.01, *** *p* < 0.001, and **** *p* < 0.0001.

As shown in Figure 1, SV40 MES 13 cells were treated with different concentrations of glucose, tamsulosin, and pioglitazone. Pioglitazone exhibited no cytotoxic effects at low concentrations. At 1 μM, cell viability was 110.12% (** *p* < 0.01); at 10 μM, viability was 109.34% (** *p* < 0.01); and, at 100 nM, viability was 106.52%. The latter concentration was selected for subsequent experiments.

Tamsulosin showed no cytotoxic effects, with maximum viability observed at 50 nM (104.55%). This concentration was chosen for further studies. For glucose, the highest viability was observed at 30 mM (115.25%, *** *p* < 0.001); meanwhile, at 20 mM, the permeability index reached 3.02, approximately threefold higher than controls (*** *p* < 0.001), indicating cellular damage comparable to that observed at 25 and 30 mM.

### 2.2. Effects of Tamsulosin and Pioglitazone on Proliferation and Viability in SV40 MES 13 Cells Activated by HG

**Figure 2:**
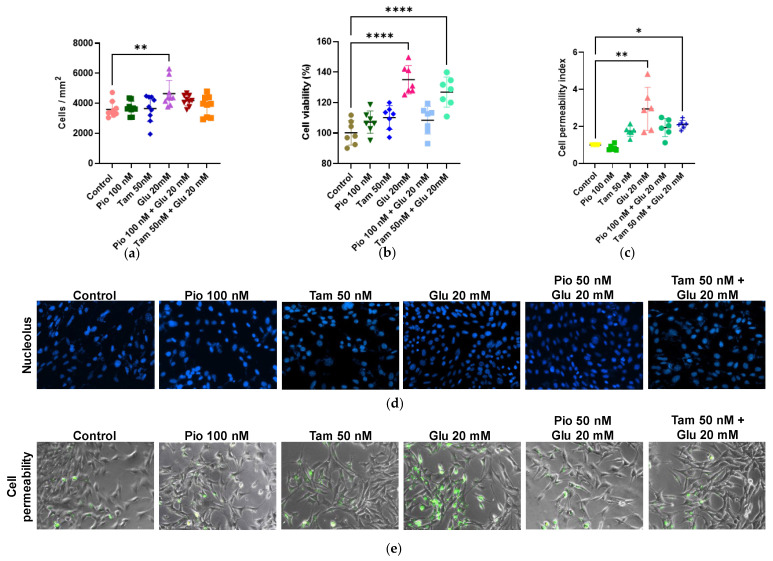
Effect of pioglitazone and tamsulosin on cell proliferation and viability in SV40 MES 13 cells. (**a**,**d**) Cell proliferation measured using the Hoechst method. Image was taken to ×200. (**b**) Cell viability was assessed using the MTT assay. (**c**,**e**) Cell permeability was detected using the SYTOX Green assay. The image was taken with ×200. Statistical significance is indicated as follows: * *p* < 0.05, ** *p* < 0.01 and **** *p* < 0.0001.

Both treatments showed no statistical difference compared to the control group (Figure 2). In contrast, cells exposed to glucose (20 mM) exhibited significantly increased proliferation (4645.78 cells/mm^2^, **** *p* < 0.0001) and viability (135.22%, ** *p* < 0.01). Furthermore, with tamsulosin and glucose, both viability (126.91%, **** *p* < 0.0001) and proliferation (3824.67 cells/mm^2^) were reduced compared to the glucose (20 mM) group (4645.78 cells/mm^2^). Nevertheless, in the glucose-treated group with pioglitazone, cell viability decreased by 108.46% with a corresponding cell proliferation of 4189.56 cells/mm^2^.

To corroborate these observations, the SYTOX Green assay was performed. The glucose (20 mM) group showed greater cell membrane permeability compared to the control group (2.95-fold, ** *p* < 0.01), indicating increased membrane damage. Tamsulosin (1.75-fold) or pioglitazone (0.83-fold) alone did not result in significant differences in cellular permeability in SV40 MES 13 cells. When tamsulosin was added to HG-exposed cells, cellular permeability decreased (2.11-fold, * *p* < 0.05). Furthermore, in the glucose group with pioglitazone, a greater decrease in cell permeability was observed, reducing it to 1.94-fold.

### 2.3. Tamsulosin and Pioglitazone Modulate α1- and β2-AR Expression Under HG Conditions

**Figure 3:**
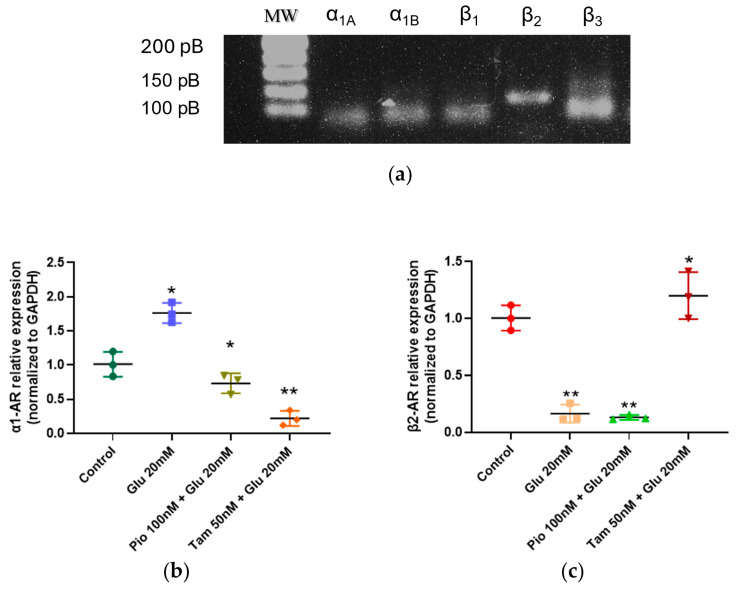
α1A-AR and β2-AR gene expression. (**a**) PCR was used to detect α1A-AR and β2-AR gene presence. RT-qPCR was used to measure the mRNA levels of (**b**) α1A-AR and (**c**) β2-AR. Statistical significance is indicated as follows: * *p* < 0.05 and ** *p* < 0.01.

We first analyzed the presence of α-AR and β-AR in SV40 MES 13 under basal conditions, as their expression had not been previously reported. As shown in Figure 3, we detected α1A-AR, α1B-AR, β1-AR, β2-AR, and β3-AR expression via PCR; then, we evaluated the effects of HG and tamsulosin (50 nM) and HG and pioglitazone (100 nM) on AR expression. Exposure to HG increased α1-AR (1.76-fold, * *p* < 0.05) and decreased β2-AR (0.12-fold, ** *p* < 0.01), compared to the control group. While treatment with tamsulosin for 24 h significantly reduced the expression of α1A-AR (0.22-fold, * *p* < 0.05), the expression of β2-AR was restored (1.20-fold, ** *p* < 0.01). On the other hand, pioglitazone treatment only slightly decreased α1-AR expression (0.73-fold, ** *p* < 0.01), while the expression of β2-AR was similar to that in the HG group, reducing its expression (0.13-fold, * *p* < 0.05).

### 2.4. Tamsulosin and Pioglitazone Attenuate HG-Induced Mitochondrial Superoxide Production and Regulate NRF2 Expression in SV40 MES 13 Cells

**Figure 4:**
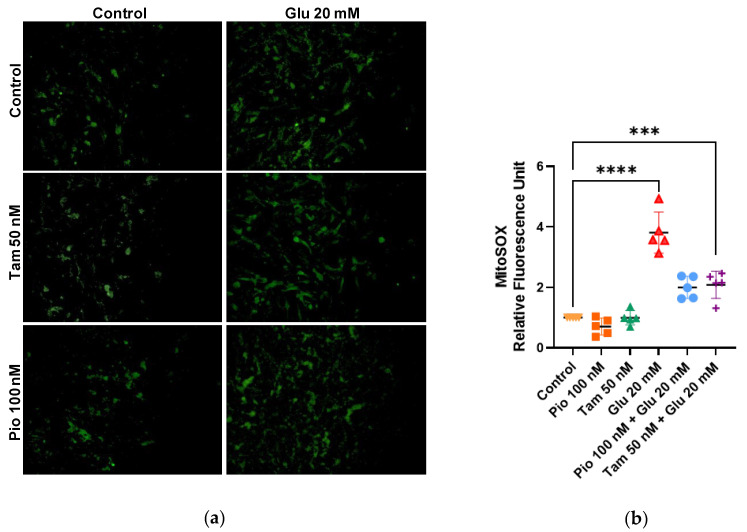
Mitochondrial superoxide ions detection. (**a**,**b**) MitoSOX assay was performed with 4 h of induction. The image was taken with ×200. Statistical significance is indicated as follows: *** *p* < 0.001 and **** *p* < 0.0001.

A MitoSOX assay was performed to detect oxidative stress through the identification of superoxide ions generated in mitochondria. In the HG group, a significantly higher quantity of ROS was observed: 3.81-fold compared to the control group (**** *p* < 0.0001). However, when tamsulosin was added, ROS levels were reduced to 2.08-fold (*** *p* < 0.001); furthermore, with pioglitazone they reduced to 2.00-fold, indicating a lower amount of superoxide ions within cells. The tamsulosin and pioglitazone groups were additionally included for comparison, where no significant differences were found with respect to the control group (Figure 4).

**Figure 5:**
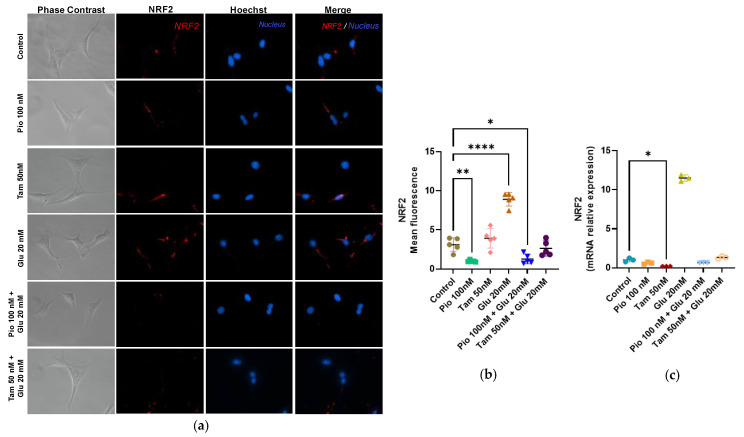
Expression of NRF2 in SV40 MES 13 cells. (**a**) Immunofluorescence detection of NRF2, using rhodamine (red) and Hoechst for the nucleus (blue). Magnification: ×200. (**b**) Summarized data of NRF2 expression as observed in the immunostaining of MCs. (**c**) NRF2 mRNA relative expression was detected by RT-qPCR, with GAPDH used as an internal control. Statistical significance is indicated as follows: * *p* < 0.05, ** *p* < 0.01 and **** *p* < 0.0001.

To analyze the cellular response to ROS, immunofluorescence was used to detect NRF2. Tamsulosin and pioglitazone presented no significant effects. MCs exposed to HG displayed a strong antioxidant response compared to the control group, with NRF2 increasing in both gene (11.51-fold) and protein expression (8.91-fold, **** *p* < 0.0001). However, the addition of tamsulosin or pioglitazone to glucose-treated cells restored NRF2 levels to values comparable to those observed in the control group (Figure 5).

### 2.5. Tamsulosin and Pioglitazone Reduced Cytoplasmic Calcium Caused by HG

**Figure 6:**
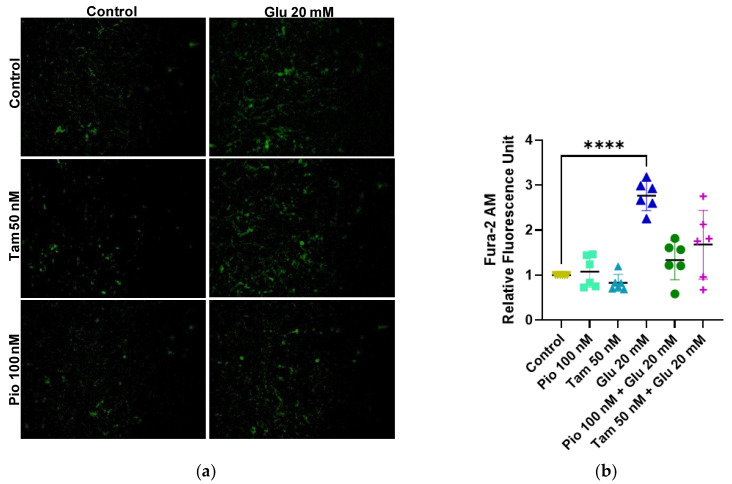
Cytoplasmic calcium levels in SV40 MES 13 cells. (**a**,**b**) Fura-2 AM dye assay was performed with 4 h of induction. The image was taken with ×200. Statistical significance is indicated as follows: **** *p* < 0.0001.

The Fura-2 AM dye assay was used to detect ionic levels of cytoplasmic free calcium. In the HG group, calcium release increased by 2.76-fold (**** *p* < 0.0001) compared to the control group. Upon addition of tamsulosin and pioglitazone, calcium release decreased to 1.68-fold and 1.34-fold, respectively, compared to the control group; however, no significant difference was found when compared with the HG-induced group (Figure 6).

### 2.6. Tamsulosin and Pioglitazone Significantly Attenuated the Inflammatory Profile in HG-Induced SV40 MES 13 MCs

**Figure 7:**
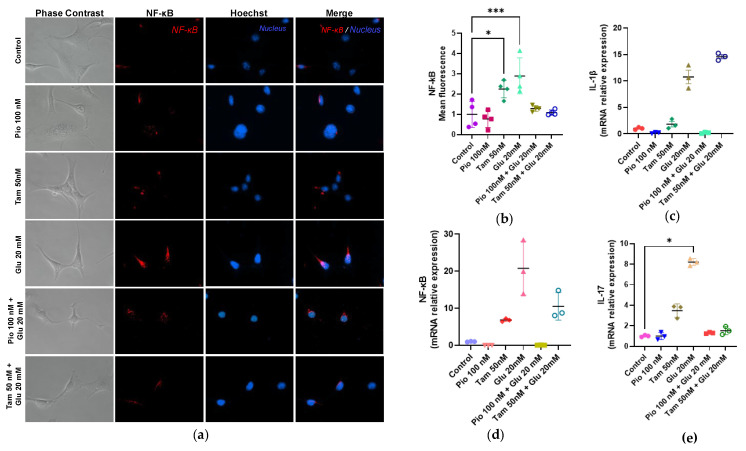
Inflammatory expression in SV40 MES 13 cells. (**a**) Immunofluorescence detection of NF-κB p65 subunit using rhodamine (red) and Hoechst for the nucleus (blue). Magnification: ×400; (**b**) Summarized data of NF-κB p65 subunit production as observed in MCs immunostaining; (**c**) IL-1β, (**d**) NF-κB, and (**e**) IL-17 mRNA relative expression was detected by RT-qPCR, with GAPDH used as an internal control. Statistical significance is indicated as follows: * *p* < 0.05 and *** *p* < 0.001.

After analyzing the results, we found that tamsulosin or pioglitazone alone did not induce an inflammatory response in MCs, as shown in Figure 7. However, in the HG-treated group, NF-κB gene expression and protein production increased by 20.78-fold (*** *p* < 0.001) and 2.89-fold (* *p* < 0.05), respectively; IL-1β increased by 10.75-fold (**** *p* < 0.0001); and IL-17 increased by 8.20-fold (**** *p* < 0.0001) compared to the control group at genetic level, indicating that HG induces a strong inflammatory profile in SV40 MES 13 MCs. When tamsulosin was combined with HG, reductions in NF-κB gene expression (10.55-fold), protein production (1.08-fold), and IL-17 expression (1.53-fold) were observed; however, tamsulosin did not reduce IL-1β expression, which instead increased to 14.60-fold (**** *p* < 0.0001). On the other hand, when pioglitazone and HG were combined, it was observed that inflammatory markers such as NF-κB were decreased at the gene and protein levels (0.08- and 1.29-fold, respectively), as well as the gene expression of IL-17 (1.30-fold) and IL-1β (0.20-fold).

### 2.7. Tamsulosin and Pioglitazone Significantly Attenuated the Fibrogenic Profile in HG-Induced SV40 MES 13 MCs

**Figure 8:**
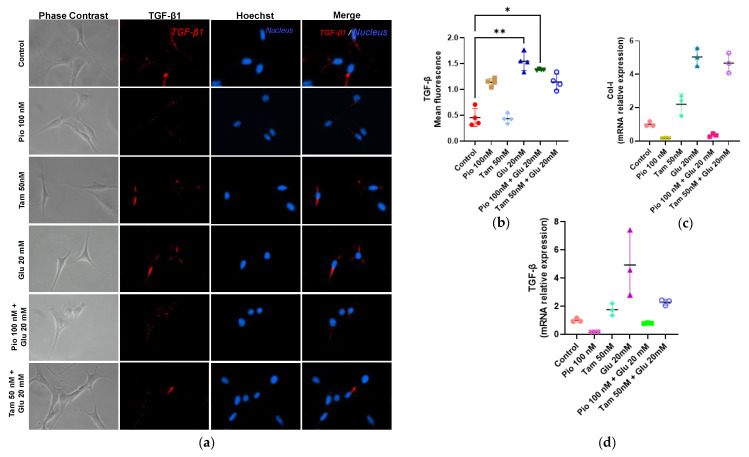
Fibrotic expression in SV40 MES 13 cells. (**a**) Immunofluorescence detection of TGF-β1, using rhodamine (red) and Hoechst for the nucleus (blue). Magnification: ×200. (**b**) Summarized data of TGF-β1 production as observed in the immunostaining of MCs. (**c**) TGF-β1 and (**d**) Col-I mRNA relative expression was detected by RT-qPCR, with GAPDH used as an internal control. Statistical significance is indicated as follows: * *p* < 0.05 and ** *p* < 0.01.

Tamsulosin or pioglitazone did not exhibit significant fibrogenic effects in MCs. However, MCs exposed to HG displayed a strong fibrogenic response compared to the control group, with increases in the gene expression of TGF-β (4.94-fold, ** *p* < 0.01) and Col I (5.04-fold, * *p* < 0.05). When tamsulosin was added in combination with HG, the expression levels of all fibrogenic markers decreased, with TGF-β (2.28-fold) and Col I (4.67-fold) being significantly reduced compared to the control group. When pioglitazone was combined with HG, it was observed that—similar to tamsulosin—TGF-β gene expression and protein production (0.79 and 1.29-fold, ** *p* < 0.01, respectively) were decreased in addition to gene expression. Furthermore, unlike tamsulosin, pioglitazone was able to decrease Col I gene expression (0.35-fold) (Figure 8).

### 2.8. Regulation of Actin Polymerization and Cellular Hypertrophy Under HG Conditions by Tamsulosin and Pioglitazone

F-actin cytoskeletal polymerization was analyzed via rhodamine–phalloidin assay. MCs in the control group showed the highest cytoskeletal activity and low α-SMA gene expression and protein production, whereas the HG group showed the lowest F-actin polymerization and increased α-SMA expression (Acta-2, 3.89-fold, * *p* < 0.05). Interestingly, tamsulosin and pioglitazone treatments in the HG group resulted in increased cytoskeleton polymerization and a reduction in α-SMA expression (*Acta-2*, 2.39-fold with tamsulosin and 0.12-fold with pioglitazone), suggesting that both treatments enhance F-actin formation through G-actin polymerization and prevent possible actin turnover, counteracting some of the damage caused by HG.

**Figure 9:**
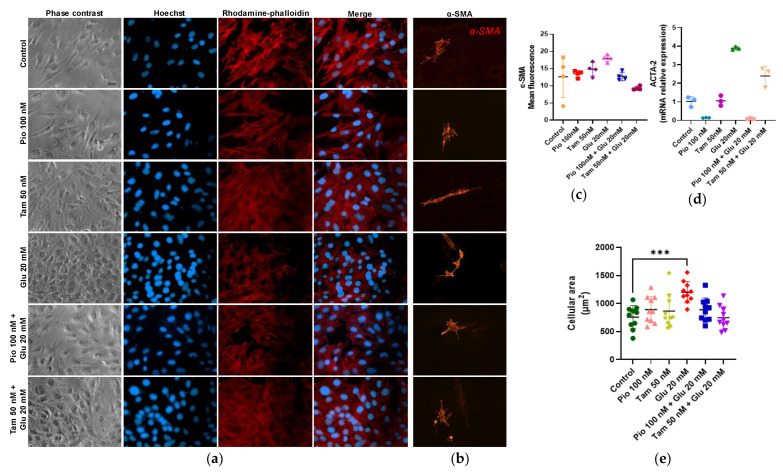
Effect of HG, pioglitazone, and tamsulosin on the cytoskeleton. (**a**) F-actin activity. Image was taken at ×400. (**b**) Immunofluorescence detection of α-SMA using Alexa Fluor 594 (red). Magnification: ×200. (**c**) Summarized data of α-SMA production as observed in MCs immunostaining; (**d**) *Acta-2* mRNA relative expression was detected by RT-qPCR, with GAPDH used as an internal control; (**e**) MCs area. Statistical significance is indicated as follows: *** *p* < 0.001.

Another factor involved in cell hypertrophy is the increase in cell area size. Therefore, cell area was measured, which revealed a statistically significant difference in cells induced with HG compared with the control group, with an average area of 1202.3 μm^2^ (*** *p* < 0.001). On the other hand, there were decreases in cells treated with pioglitazone or tamsulosin, with average areas of 888.6 and 746.8 μm^2^, respectively (Figure 9).

### 2.9. Tamsulosin and Pioglitazone Diminished Mesangial Expansion and Positive Cells for α-SMA in an Experimental DN Model

**Figure 10:**
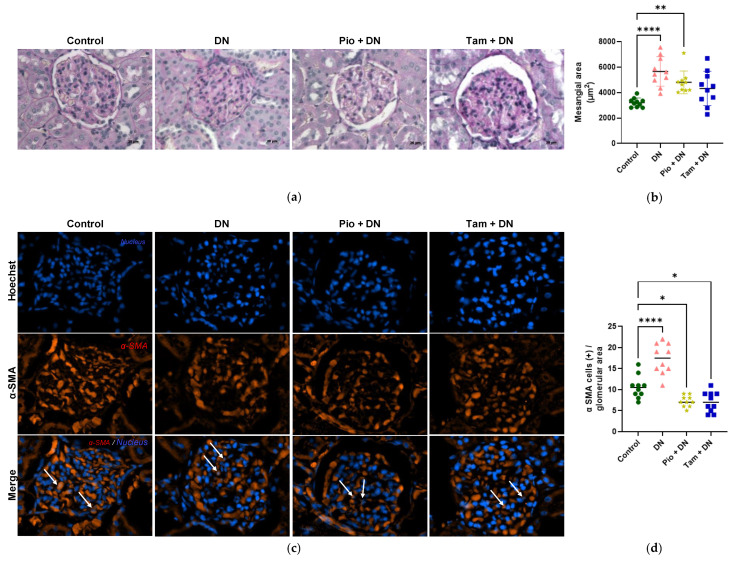
Effect of tamsulosin and pioglitazone on mesangial expansion in Wistar rat tissue. (**a**) PAS staining. Image was taken at ×400. (**b**) Summarized data of mesangial area within the glomerulus. (**c**) The white arrows indicate MCs positive for α-SMA. Immunofluorescence detection of α-SMA using Alexa Fluor 594 (red) and Hoechst (blue). Magnification: ×400. (**d**) Summarized data of α-SMA production as observed in glomerulus immunostaining. Statistical significance is indicated as follows: * *p* < 0.05, ** *p* < 0.01 and **** *p* < 0.0001.

To measure mesangial expansion in vivo, PAS staining was performed to distinguish MCs and mesangial matrix in the glomerulus according to PAS positivity. Figure 10 shows that the control group had a glomerular mesangial mean area of 3219.3 μm^2^, while rats with DN had a mean of 5659.7 μm^2^, which is a highly significant difference (**** *p* < 0.0001). On the other hand, rats treated with pioglitazone showed a mean glomerular mesangial area of 4807.8 μm^2^ (** *p* < 0.01) while those treated with tamsulosin obtained 4324.1 μm^2^. Therefore, the treatments decreased the mesangial area, reducing the mesangial expansion caused by induced hyperglycemia.

Furthermore, immunofluorescence was performed to detect α-SMA-positive cells, demonstrating that rats that developed DN had a higher number of positive cells, with an average of 17.3 (**** *p* < 0.0001) positive cells per glomerulus; in contrast, this value decreased to 7.2 (* *p* < 0.05) in the group treated with pioglitazone and 7.1 (* *p* < 0.05) in the tamsulosin group, indicating that these treatments possibly reduced the number of CMs transdifferentiated into myofibroblasts.

## 3. Discussion

CKD is a long-term complication of DM caused by the sustained hyperglycemia that patients suffer [40], with complications characterized by vascular alterations and metabolic complications due to changes in the extracellular matrix. It has repercussions for the eyes (retina), peripheral vascularization (diabetic foot), and highly irrigated organs. Specifically, it induces mesangial expansion and extracellular matrix accumulation in the kidneys, leading to alterations in glomerular physiology [41,42,43].

HG medium has been widely reported in in vitro experiments as a model of DN, typically used at concentrations of 20, 25, and 30 mM [44,45]. However, some studies have reported cytotoxicity at 30 mM [46]. We found that exposing MCs to these three glucose concentrations increased proliferation and cell hypertrophy, as well as cell permeability; cell damage was observed in DN. As the 20 mM concentration was associated with increased proliferation and permeability in all replicates, it was used in the rest of the analyses. In addition, converting 20 mM to mg/dL yielded a glucose concentration of 360.31 mg/dL, which is an above-normal concentration in an uncontrolled diabetic patient.

One of the primary challenges in this context is the absence of highly effective treatments, largely due to the complexity of the underlying inflammatory and fibrogenic mechanisms in DN [26,47]. First-line therapy typically involves medications aimed at controlling hyperglycemia, including iSGLT2 and GLP-1 receptor agonists [27], which are commonly used in patients with proteinuria or hypertensive nephropathy. However, they mainly serve to slow the progression of these diseases, often necessitating high doses to achieve a therapeutic benefit which, in turn, can elevate the risk of adverse effects [26,47].

One of the aims of this work was to evaluate the role of AR regulation on glucose metabolism in MCs, in order to avoid its activation in a HG medium—a scenario observed in patients with DN. There have been several reports on the effects of stimulation of α- or β-AR on MCs; for example, the stimulation of α1-AR generates a contractile effect, increases extracellular matrix synthesis, and elicits a pro-inflammatory response [42,48]. Stimulation of β-AR on MCs—specifically β2—regulates the immune response, increases mitochondrial function and biogenesis, and optimizes the function of the respiratory chain [29].

For this study, we initially demonstrated that SV40 MES 13 MCs express different α- and β-AR subtypes, as found in primary cultures of MCs. Based on Figure 1, α1-, α2-, β-, β1-, β2-, and β3-AR were found to be expressed on MCs.

Tamsulosin—an α1A- and α1D-AR antagonist [49]—is commonly administered to treat benign prostatic hyperplasia. Nevertheless, there are very few studies about its renoprotective effects and its possible benefits with respect to the control of fibrogenic processes observed in DN.

In relation to the behaviors of α1- and β2-AR in the SV40 MES 13 cell line incubated in HG medium, we observed a change in the expression of these receptors: the expression of α1-AR increased, while that of β2-AR decreased. This is similar to the findings of Safi et al. (2014) [50] and Papay et al. (2020) [51], who reported that β-ARs are affected in endothelial cells, while α-ARs show increased expression. Thus, our results suggest that increased α1-AR expression in mesangial cells promotes their activation, leading to a pro-inflammatory and profibrogenic state [50,51]. When adding tamsulosin, α-AR decreased while β-AR was restored, indicating a possible regulatory effect on activation of these cells. Pioglitazone attenuated the HG-induced increase in α1-AR, but did not restore β2-AR expression. This suggests that β2-AR downregulation is primarily driven by hyperglycemia and occurs independently of PPARγ-mediated pathways.

In the mesangium, mesangial expansion and increased mesangial matrix production occur as a result of MC activation [19]. For this reason, viability and proliferation tests were performed to determine whether the MCs were activated. Tamsulosin or pioglitazone were not capable of modifying the proliferation status or viability of MCs, thus indicating a lack of MC activation. To the contrary, in HG medium, MC proliferation was increased, suggesting their activation. A higher number of membrane-damaged cells was observed in the HG group via Sytox Green assay; a similar effect has been reported by Kang et al. (2003), which was attributed to HG medium inducing apoptosis in cells due to ROS generation [47]. The generation of ROS was also observed in our work, with the amount of superoxide increasing in a greater proportion when MCs were induced with HG. When combining HG with tamsulosin or pioglitazone, the amount of damaged cells decreased, demonstrating that both treatments reduce the damage caused by HG; possibly due to the regulation of mitochondrial function.

Regulation of the cellular antioxidant process is controlled by the Nuclear Factor erythroid 2 Related Factor 2 (NRF2) system which, in turn, is regulated by Keap 1 protein inhibition [52]. The main oxidative factors are ROS generated in mitochondria, which are produced during the cellular respiratory process under HG conditions [53].

Under a HG intracellular concentration, the polyol pathway is also activated and decreases the availability of NADPH—a cofactor necessary to regenerate reduced glutathione, which acts as an antioxidant within cells [11]. It is possibly for this reason that an increase in antioxidant systems was observed in MCs, due to an increase in ROS production after exposure to the HG medium. In addition, exposure to oxidative stress leads to the overproduction of pro-inflammatory cytokines (e.g., IL-1β) which, in turn, amplifies oxidative stress and further activates NF-κB, establishing a positive feedback loop of inflammation. Activation of the NRF2/antioxidant response elements (ARE) system interrupts this cycle by inducing antioxidant and anti-inflammatory genes—including heme oxygenase-1 (HO-1)—which mitigate oxidative damage and limit the secretion of pro-inflammatory cytokines and chemokines. In turn, NF-κB activity influences the Keap1/NRF2/ARE pathway through multiple mechanisms: Keap1 mediates the ubiquitination and degradation of inhibitor of nuclear factor kappa-B kinase (IKKβ), thereby inhibiting NF-κB; inflammatory mediators such as cyclooxygenase 2 (COX-2) react with Keap1 to activate NRF2, coordinating gene transcription while simultaneously suppressing NF-κB activity; and NF-κB competes with NRF2 for the transcriptional co-activator cAMP-response-element-binding protein (CBP). Collectively, these mechanisms highlight the reciprocal regulation between NRF2 and NF-κB as a key modulator of oxidative stress-induced inflammation [52,54].

The increased NRF2 gene and protein expression observed in MCs may result from HG exposure. Although NRF2 is generally cytoprotective, excessive activation has been linked to pro-apoptotic effects. In pancreatic β-cells, Keap1 overexpression—which suppresses NRF2—restored proliferation and reduces apoptosis [55]. Similar pro-apoptotic effects of NRF2 have been reported in renal cells, through interactions with p53 and mitochondrial pathways [56].

Tamsulosin induced decreases in the expression of superoxide and NRF2, which are markers of oxidation. This was also observed, in the study by Sun et al. (2021) [31], in endothelial cells treated with tamsulosin. These results suggest that this drug has an antioxidant function, preventing NF-κB activity and, therefore, reducing ROS levels, thus resulting in decreases in inflammatory (NF-κB, IL-6 and IL-8) and fibrogenic (TGF-β1 and Col I) markers. In our study, tamsulosin decreased NF-κB and TGF-β expression in MCs, suggesting that—similar to glomerular endothelial cells—tamsulosin inhibits NF-κB, thus exerting an anti-inflammatory effect and leading to decreased fibrosis [31]. MCs exposed to HG conditions and treated with tamsulosin showed increased IL-1β expression, despite a reduction in NF-κB levels. This paradox may result from NF-κB-independent pathways, such as those involving AP-1 and MAPKs [57,58]. These findings suggest that, although tamsulosin suppresses classic inflammatory mediators such as NF-κB and IL-17, IL-1β expression is maintained through alternative transcriptional mechanisms, which could modulate the contractile and proliferative activities of these HG-induced cells. As there are few studies that have discussed the associated anti-inflammatory and anti-fibrogenic activities, more studies focused on the signaling pathways involved should be conducted.

The anti-inflammatory and anti-fibrogenic effects of pioglitazone have been previously reported in in vivo models [36,59]. In the present work, it was also observed that pioglitazone has anti-inflammatory and anti-fibrotic effects in MCs; nevertheless, limited research has been conducted on MCs to date, and further in vitro studies are required to understand the signaling pathways involved.

On the other hand, intracellular calcium is related to numerous cellular functions [60]. In MCs, it is mainly related to cell contraction [61,62]; although, under pathological conditions, an increase in intracellular calcium can lead to apoptosis [60] or necrosis, damaging the mitochondria or endoplasmic reticulum [63]. Nevertheless, few studies have examined the effects of high intracellular Ca^2+^ concentrations in MCs induced by HG. In a study published by Saleh et al. (2000), the authors demonstrated that HG concentrations increased intracellular Ca^2+^, initiating the apoptosis signaling pathway [64]. In contrast, both treatments reduced calcium release, suggesting that these treatments reduce cell damage due to HG levels.

We also evaluated the behaviors of cytoskeleton components. It has been reported that, in fibroblast lines, F-actin decreases while the expression of α-SMA increases when stimulated with TGF-β, leading to their differentiation into myofibroblasts; it follows that, in this work, there was a possible phenotypic change in the type of actin found in myofibroblasts [65].

Based on these findings in fibroblasts we observed that, upon interaction with HG medium, MCs presented decreased polymerization of G-actin to F-actin, as well as increased protein and gene expression of α-SMA, actin fibers, sclerostin, and cellular stress fibers; furthermore, the response of calcium channels increased in response to the HG medium, which correlates with the possible phenotypic change in the cytoskeleton, as this results in the increased expression of contractile fibers. These outcomes demonstrate that the considered cells were strongly activated by the HG medium, corroborating results previously obtained in relation to pro-inflammatory and pro-fibrogenic responses observed in these cells [66]. To confirm the in vitro findings, an in vivo study was conducted using immunofluorescence to detect *α*-SMA production in MCs. As a result, we suggest that the treatments reduced the transdifferentiation of MCs caused by HG. Previous studies have reported the anti-fibrotic and anti-inflammatory effects of tamsulosin in liver and lung injury models [67,68]; however, its impact on MCs under HG conditions had not previously been thoroughly investigated. This study demonstrates that tamsulosin modulates the expression of ARs, pro-inflammatory cytokines (IL-1β, IL-17, NF-κB), and fibrotic markers (α-SMA, Col-1), suggesting its potential roles in reducing hyperglycemia-induced stress.

Notably, tamsulosin did not significantly reduce Col-1 expression in MCs under HG conditions (HG: 5.04-fold; HG and tamsulosin: 4.67-fold), indicating minimal impact compared with the HG group. This may reflect the ability of MCs to adopt distinct phenotypes in response to stimuli: quiescent cells that maintain glomerular homeostasis, activated cells expressing inflammatory and fibrogenic markers, and inactivated cells that retain a semi-activated state with residual α-SMA and collagen expression but no longer drive pathology [66]. Tamsulosin may facilitate a shift toward this inactivated phenotype under HG conditions, modulating mesangial activation without fully suppressing fibrogenic markers.

In addition to the information mentioned above, the organization of the cytoskeleton, the presence of F-actin, and the increase in the cell area of MCs provide information on whether the cell is hypertrophied, which causes disorganization of the cytoskeleton (actin fibers), an increase in cell area, and a lower presence of F-actin [66,69,70]. Therefore, glucose was confirmed to cause cell hypertrophy, which has already been widely reported [71,72]; however, this study demonstrates for the first time that both tamsulosin and pioglitazone reduce the cell hypertrophy in MCs caused by exposure to HG.

Finally, an in vivo study was conducted to corroborate the behavior of the mesangium in streptozotocin-induced diabetic rats and the effects of both treatments on mesangial expansion. Using the PAS staining technique, we observed an increase in the mesangial area, indicating mesangial expansion associated with high blood glucose concentrations. This finding is consistent with our in vitro study, where MCs exhibited an increase in cell size and mesangial matrix secretion under HG conditions. Notably, treatment with either pioglitazone or tamsulosin significantly reduced the mesangial area. These results suggest that both drugs may help to mitigate the mesangial expansion induced by hyperglycemia. This finding is consistent with the study conducted by Morones J. et al. (2024) [39] in our laboratory, demonstrating that both treatments increased the estimated glomerular filtration rate (eGFR) in an in vivo model of DN. Moreover, they reduced serum damage markers including fasting blood glucose (FBG), serum creatinine, blood urea nitrogen (BUN), and urea levels, as well as urinary markers of renal injury such as glucose, BUN, and urea, while increasing urinary creatinine. In addition, histopathological analysis revealed decreases in markers of inflammation and fibrosis, indicating that both treatments exerted a renoprotective effect [39].

In conclusion, our results demonstrate that both tamsulosin and pioglitazone attenuate the activation of MCs induced by HG exposure by mitigating oxidative stress, cell proliferation, and hypertrophy. It should be noted that pioglitazone showed superior efficacy compared to tamsulosin, as it significantly reduced the gene and protein expression of key inflammatory markers as well as TGF-β, SMA, and collagen I—critical indicators of MC activation. In in vivo experiments, both treatments effectively limited mesangial expansion and activation. Taken together, these findings suggest that tamsulosin and pioglitazone may serve as protective agents against the activation of MCs and glomerular remodeling.

## 4. Materials and Methods

### 4.1. Animals

A total of 20 male Wistar rats (6 ± 1 weeks old, 180–200 g) from the Universidad Autónoma de Aguascalientes were housed under sterile, pathogen-free conditions with controlled light cycles (12 h light/dark cycles), humidity (50–60%), and temperature (20–25 °C). They received a standard rodent diet supplemented with 50 g of food (Purina^®^ DOG CHOW mature age, Nestlé, Mexico City, Mexico) and water with 1% sucrose, both provided ad libitum. The experimental protocol was approved by the UAA Ethics Committee (CEADI-UAA, UAA: Universidad Autónoma de Aguascalientes, AUT-BC-1121-077-Type C) following the Mexican Official Standard NOM-062-ZOO-1999 [73], following national regulations and NIH guidelines [74].

After a one-week acclimatization, diabetes was induced using a single intraperitoneal dose of streptozotocin (STZ) (50 mg/kg, Sigma Aldrich Biotechnology, St. Louis, MO, USA) [75] dissolved in a freshly prepared 0.1 mol/L citrate buffer solution at pH 4.5), 3 days after the STZ injection were diagnosed as diabetic [76].

Rats presenting fasting glucose levels above 13.9 mmol/L three days post-injection were classified as diabetic. The animals were randomly divided into four groups (n = 5): non-diabetic control, diabetic control (STZ-induced), diabetic group treated with tamsulosin (0.4 mg/kg, dissolved in purified water and administered orally, Pharmalife, Jalisco, Mexico), and diabetic group treated with pioglitazone (60 mg/kg, suspended in carboxymethylcellulose and given orally, Pharmalife, Jalisco, Mexico). Six weeks after diabetes induction, the rats were euthanized with an intraperitoneal injection of sodium pentobarbital (≥100 mg/kg), and kidney tissues were collected for further analysis.

#### 4.1.1. Mesangial Expansion Measurement

Following a 2-day fixation period in 10% neutral buffered formalin, kidney samples were subjected to a graded alcohol dehydration protocol (96%, 96%, 100%, 100%), each step lasting 1 h. This was followed by tissue clearing in xylene (two 1 h treatments) and paraffin embedding through two 2 h infiltration steps. Sections of 5 μm thickness were obtained and stained using periodic acid–Schiff (PAS) stain. Image was performed using a CoolSNAP-Pro camera (ZEISS, Göettingen, Germany) attached to a ZEISS Axioskop 40 microscope (ZEISS, Göettingen, Germany). Mesangial area was measured using Fiji Is Just, v1.54f software (Wayne Rasband, National Institutes of Health, Bethesda, MD, USA).

#### 4.1.2. Mesangial α-SMA by Immunofluorescence

Tissue samples preserved in paraformaldehyde were cut into 1 cm^3^ fragments and sequentially immersed in 15% and then 30% sucrose solutions, each for 24 h. Subsequently, 5 µm cryosections were prepared using a cryostat (HYRAX C 2′, ZEISS, Walldorf, Germany) and placed on a hot plate for 5 min to enhance adherence. Sections were rinsed in for 10 min.

To block nonspecific binding, slides were incubated with 10% fetal bovine serum diluted in PBS 1X at 37 °C for 30 min in a wet chamber.

For immunofluorescence staining, primary antibodies were applied at a 1:100 dilution in PBS 1X and incubated overnight at 4 °C in a humid chamber. The antibody used was α-SMA (1:100, Sigma-Aldrich, A5228, St. Louis, MO, USA). Following three washes of 5 min in PBS 1X, a secondary antibody—anti-rabbit IgG conjugated with Alexa Fluor (Life technologies, A11005, Eugene, OR, USA) was applied at a 1:1000 dilution in PBS 1X, incubated for 2 h at room temperature in the dark within a wet chamber. Nuclei were counterstained using Hoechst 33258 for 10 min. Finally, slides were mounted using Glicergel (Dako, Glostrup, Denmark, C0563). Fluorescence detection was performed using an Axiovert 40 CFL microscope (ZEISS, Göttingen, Germany) attached to a ZEISS Axiovert 40 CFL camera (ZEISS, Göettingen, Germany) with filters set to an excitation wavelength of 546/12 (red) and 365 nm (blue).

### 4.2. Cell Culture

SV40 MES 13 (ATCC, CRL-1927, Manassas, VA, USA) was donated by the immunology Laboratory of the National School of Biological Sciences, Instituto Politécnico Nacional (IPN). Cells were maintained at 37 °C with 5% CO_2_ and 100% humidity in DMEM/Ham’s F12 medium (50/50 mixture) with L-glutamine (Corning, 10-090-CV, Christiansburg, VA, USA), supplemented with 5% fetal bovine serum (FBS, Sigma-Aldrich, F4135, St. Louis, MO, USA), as well as penicillin (1000 U/mL) and streptomycin (1000 μg/mL) (Caisson, ABL02, Smithfield, UT, USA). For propagation, once cells reached 80% confluence, they were detached using 0.25% (*w*/*v*) trypsin-EDTA (Sigma-Aldrich, T4049, St. Louis, MO, USA) and transferred to a T-25 flask, seeding 15 × 10^3^ cells. The culture medium was changed every 72 h.

#### 4.2.1. Colorimetric MTT (Tetrazolium) Viability Assay

Thiazolyl Blue Tetrazolium Bromide (MTT) assay was performed to examine viability. SV40 MES 13 (3 × 10^3^ cells/well) were seeded in 96-well plates and then they were incubated with D-(+)glucose (20 mM, Sigma-Aldrich, G7021, St. Louis, MO, USA) and/or tamsulosin hydrochloride (50 nM, Sigma-Aldrich, T1330, St. Louis, MO, USA) or pioglitazone (100 nM, Sigma Aldrich, E6910, St. Louis, MO, USA) for 24 h. Afterwards, 20 μL of MTT solution (5 mg/mL, Sigma-Aldrich, M5655, St. Louis, MO, USA) was added to each well to incubate them for 4 h. Subsequently, 100 μL of DMSO (Sigma Aldrich, D1435, St. Louis, MO, USA) was added after discarding the supernatant to dissolve MTT-formazan crystals. Finally, absorbance was measured using a microplate reader at a wavelength of 490 nm (BIO-RAD, Hercules, CA, USA). The concentrations of tamsulosin (50 nM) and pioglitazone (100 nM) were selected based on these viability assays and prior literature, reflecting clinically relevant and pharmacologically active in vitro levels [67,77,78,79,80,81].

#### 4.2.2. SYTOX Green Membrane Cell Permeability Determination by Using High Glucose Medium

SV40 MES 13 (3 × 10^4^ cells/well) was grown and exposed to glucose (20 mM) and/or tamsulosin (50 nM) or pioglitazone (100 nM) for 24 h in 24-well plates. Subsequently, they were washed briefly with PBS 1X and fixed with 4% paraformaldehyde for 10 min and incubated with SYTOX green (60 mM, Invitrogen, S7020, Eugene, OR, USA) for 17 min at 37 °C to detect nucleic acid staining. Fluorescence detection was performed using an Axiovert 40 CFL microscope (ZEISS, Göttingen, Germany) attached to a ZEISS Axiovert 40 CFL camera (ZEISS, Göettingen, Germany) with a filter set to an excitation wavelength of 450–490 nm (green).

#### 4.2.3. Detection of Mitochondrial Superoxide Formation by MitoSOX Assay

SV40 MES 13 was exposed to glucose (20 mM) and/or tamsulosin (50 nM) or pioglitazone (100 nM) for 4 h in 24-well plates. They were washed briefly with PBS 1X, fixed with 1% paraformaldehyde for 10 min at room temperature and incubated 1 h at 37 °C with MitoSOX green (2 μM, Invitrogen, M36006, Eugene, OR, USA). Fluorescence detection was performed using an Axiovert 40 CFL microscope (ZEISS, Göttingen, Germany) attached to a ZEISS Axiovert 40 CFL camera (ZEISS, Göettingen, Germany) with a filter set to an excitation wavelength of 450–490 nm (green). Quantification of fluorescence intensity of mitochondrial superoxide was performed using Fiji Is Just, v1.54f software (Wayne Rasband, National Institutes of Health, Bethesda, MD, USA). Initially, images were converted to 8-bit greyscale and fluorescence intensity was measured and expressed as relative fluorescence unit, defining them as the threshold for all pixels within the indicated area.

#### 4.2.4. Intracytoplasmic Ca^2+^ Measurement

SV40 MES 13 (3 × 10^4^ cells/well) was induced with glucose (20 mM) and/or tamsulosin (50 nM) or pioglitazone (100 nM) for 24 h in 24-well plates, afterwards, 1% paraformaldehyde was used to fix them for 10 min. Following, these cells were incubated with Fura-2 AM (1 μM, Invitrogen, F1201, Eugene, OR, USA) for 30 min at 37 °C and washed with PBS 1X. Fluorescence detection was performed using an Axiovert 40 CFL microscope (ZEISS, Göttingen, Germany) attached to a ZEISS Axiovert 40 CFL camera (ZEISS, Göettingen, Germany) with a filter set to an excitation wavelength of 450–490 nm (green). Quantification of fluorescence intensity of cytoplasmatic calcium was measured using the Fiji Is Just, v1.54f software (Wayne Rasband, National Institutes of Health, Bethesda, MD, USA). Images were converted to 8-bit greyscale and fluorescence intensity was measured and expressed as relative fluorescence unit, defining them as the threshold for all pixels within the indicated area.

#### 4.2.5. Polymerized Actin Detection Assay by Identification of Transformation from G-Actin to F-Actin

SV40 MES 13 (3 × 10^4^ cells/well) was grown and induced for 24 h in 24-well plates with glucose (20 mM) and/or tamsulosin (50 nM) or pioglitazone (100 nM). Subsequently, they were fixed with 4% paraformaldehyde for 10 min. Following, a wash with PBS 1X they were incubated with rhodamine-phalloidin (6.6 μM, Invitrogen, R415, Eugene, OR, USA) at room temperature for 30 min to detect F-actin. Then, nuclei were counterstained with Hoechst 33258 (1:1000, Sigma-Aldrich, 94403, St. Louis, MO, USA). Fluorescence detection was performed using an Axiovert 40 CFL microscope (ZEISS, Göttingen, Germany) attached to a ZEISS Axiovert 40 CFL camera (ZEISS, Göettingen, Germany) with filters set to an excitation wavelength of 546/12 (red) and 365 nm (blue).

#### 4.2.6. Immunofluorescence

SV40 MES 13 (3 × 10^4^ cells/well) was grown on coverslips treated with poly-L-Lysine (Sigma Aldrich, P4707, St. Louis, MO, USA) in culture dishes, and they were washed briefly with PBS 1X and fixed with methanol for 10 min at 4 °C. After being washed, cells were blocked with 1% SFB and incubated with primary antibodies (1:1000) TGF-β1 (Novus, NBP1-03276, Centennial, CO, USA), NF-κBp65 (Invitrogen, 44-711G, Bengaluru, Karnataka, India), NRF2 (Cell Signaling Technology, D1Z9C, MA, USA) or α-SMA mouse monoclonal anti-mouse antibody (Sigma-Aldrich, A5228, St. Louis, MO, USA) for 24 h at 4 °C. MCs were incubated with fluorescent conjugated secondary antibody (1:1000) rhodamine goat anti-rabbit IgG (H+L) (1:1000, Invitrogen, R-6394, Eugene, OR, USA), for the α-SMA antibody Alexa fluor 594 (1:1000, Life technologies, A11005, Eugene, OR, USA) was used and nuclei were counterstained with Hoechst 33258 (1:1000). Finally, slides were mounted using Glicergel (Dako, Glostrup, Denmark, C0563). Fluorescence detection was performed using a CoolSNAP-Pro camera (ZEISS, Göettingen, Germany) attached to a ZEISS Axioskop 40 microscope (ZEISS, Göettingen, Germany) with filters set to an excitation wavelength of 546/12 (red) and 365 nm (blue).

#### 4.2.7. Mesangial Cell Area Measurement

SV40 MES 13 (3 × 10^4^ cells/well) was grown and induced for 24 h in 24-well plates with glucose (20 mM) and/or tamsulosin (50 nM) or pioglitazone (100 nM). Subsequently, they were fixed with 4% paraformaldehyde for 10 min. Following, a wash with PBS 1X. MCs was taken used phase contrast using an Axiovert 40 CFL microscope (ZEISS, Göttingen, Germany) attached to a ZEISS Axiovert 40 CFL camera (ZEISS, Göettingen, Germany).

#### 4.2.8. Analyze Gene Expression by RT-qPCR

After 24 h of cell induction with glucose (20 mM) and/or tamsulosin (50 nM) or pioglitazone (100 nM). SV40 MES 13 (3 × 10^5^ cells/well) was treated with Direct-zol™ RNA MiniPrep^®^ kit (Zymo Research, R2052, Irvine, CA, USA), following manufacturer’s instructions. Total RNA was quantified using Biodrop (Isogen Life Science, Utrecht, The Netherlands) and it was stored at −80 °C until used. Reverse transcription was performed with 1 µg of total RNA using the RevertAid First-Strand cDNA Synthesis kit (Thermo Scientific, K1621, Plainville, MA, USA). Subsequently, qPCR was performed using StepOne (Applied Biosystems, Eugene, MA, USA) and Maxima SYBR Green/ROX qPCR Master Mix (2×) (Thermo Scientific, K0221, Plainville, MA, USA) under the following conditions: at 50 °C for 2 min, 95 °C for 3 min, followed by 40 cycles of 95 °C for 45 s and 58 °C for 40 s (The oligonucleotides used are shown in Table 1). Relative expression was normalized against GAPDH as a constitutive gene and differences were determined using the relative ΔΔCt method.

### 4.3. Statistical Analysis

Statistical analysis was performed using GraphPad Prism 9.4.1 (GraphPad, San Diego, CA, USA). Data were presented as the mean ± standard deviation (SD) for each group. Differences among the control and other distinct groups were determined using a one-way analysis of variance (ANOVA), followed by either the Kruskal–Wallis or Dunnett’s post-test. *p* values ≤ 0.05 were considered statistically significant.

## Figures and Tables

**Table 1:** Oligonucleotides sequences used for RT-qPCR.

Genes	Sequences
*IL-1β*	Fw: 5′-TGCCACCTTTTGACAGTGATG-3′
	Rv: 5′-AAGGTCCACGGGAAAGACAC-3′
*IL-17A*	Fw: 5′-CCCCTTCACTTTCAGGGTCG-3′
	Rv: 5′-GGGGGTTTCTTAGGGGTCAG-3′
*NRF2*	Fw: 5′-CTAAAGCACAGCCAGCACAT-3′
	Rv: 5′-TGTCTTTTGTGAATGGGGCTTT-3′
*NF-κB*	Fw: 5′-ACTGGAGTTGTACGGCAGTG-3′
	Rv: 5′-TGTAAAATGCATAAAACGGGGAAA-3′
*Acta-2*	Fw: 5′-GTACCCAGGCATTGCTGACA-3′
	Rv: 5′-GAGGCGCTGATCCACAAAAC-3′
*Col I*	Fw: 5′-GGGGCAAGACAGTCATCGAA-3′
	Rv: 5′-GAGGGAACCAGATTGGGGTG-3′
*TGF-β1*	Fw: 5′-ACTGGAGTTGTACGGCAGTG-3′
	Rv: 5′-GGGGCTGATCCCGTTGATTT-3′
*ADRA1A*	Fw: 5′-GGTCTGCTAGAGCCATCCTG-3′
	Rv: 5′-TGATCTTGGTGTCTGCAAGC-3′
*ADRA1B*	Fw: 5′-CGCCCACCAACTACTTCATT-3′
	Rv: 5′-AATGGAGATGGCACATAGGC-3′
*ADRB1*	Fw: 5′-CGGCCTTTCGTGTGTTTAAT-3′
	Rv: 5′-CACACCAAACCTGAGCTGAA-3′
*ADRB2*	Fw: 5′-TGGTTGGGCTACGTCAACTC-3′
	Rv: 5′-TCCGTTCTGCCGTTGCTATT-3′
*ADRB3*	Fw: 5′-GACAGCCTCAAATGCATCCT-3′
	Rv: 5′-CCCAGTCCACACACCTTTCT-3′
*GAPDH*	Fw: 5′-AGTCTACTGGCGTCTTCACC-3′
	Rv: 5′-CCACGATGCCAAAGTTGTCA-3′

## Data Availability

The data presented in this study are available on request from the corresponding author due to privacy.
